# The mediating effect of air pollution on the association between meteorological factors and influenza-like illness in China

**DOI:** 10.1186/s12889-025-21651-5

**Published:** 2025-02-08

**Authors:** Qinling Yan, Robert A. Cheke, Sanyi Tang

**Affiliations:** 1 School of Science, Chang’an University, Middle-section of Nan’er Huan Road, Xi’an, 710064 ShaanXi Province P.R. China; 2 Natural Resources Institute, University of Greenwich at Medway, Chatham Maritime, Kent, ME4 4TB England UK; 3 School of Mathematical Sciences, Shanxi University, Wucheng Road, Taiyuan, 030006 ShanXi Province P.R. China

**Keywords:** Mediating effect, Model selection, Air pollution, Meteorological factors, Influenza-like illness (ILI)

## Abstract

**Purpose:**

Although numerous studies have explored the complex relationship between air pollution, meteorological factors and respiratory infections, evidence for a mediating effect of air pollutants being involved in the association between meteorological factors and Influenza-like illness (ILI) is limited.

**Methods:**

Correlations among ILI cases, air pollutants and meteorological factors were examined with Pearson correlation analyses. Further, we formulated six candidate mediation models to explore the mediating effect of air pollutant on the association between meteorological factors and ILI infections.

**Results:**

The meteorological factors minimum temperature/maximum humidity moderated by maximum humidity/minimum temperature and pressure directly affect ILI infections, and that some of meteorological factors can also indirectly affect them through air pollutants. Increases in maximum humidity and minimum temperature can directly reduce the numbers of ILI cases, or indirectly reduce them by reducing the concentration of air pollutants.

**Conclusion:**

When the haze with low temperature, low humidity is forecasted by the meteorological agency, the environmental protection departments can take effective control measures to reduce the concentration of air pollutants, and public health departments should advocate human behavioral changes in order to mitigate and control ILI prevalence.

**Supplementary Information:**

The online version contains supplementary material available at 10.1186/s12889-025-21651-5.

## Introduction

Industrialization and urbanization and the ensuing air pollution has led to major public health issues in many countries. According to a WHO report, about 7 million people die each year due to exposure to fine particles in the polluted air that can penetrate into the cardiovascular system and lungs [1]. As the largest and most populous developing country in the world, China was experienced particularly serious air pollution issues in the past few years [2–8].

Emerging toxicological and epidemiological studies have shown that, in addition to its adverse effects on visibility and global climate [2, 3], air pollutants can also induce respiratory diseases such as bronchial asthma, chronic bronchitis, obstructive emphysema, chronic obstructive pulmonary disease [9], cardiovascular diseases such as acute (slow) heart disease, myocardial infarction, hypertension and cerebral hemorrhage as well as increasing the incidence of infectious diseases [8].

Amongst, respiratory diseases, Influenza-like illness (ILI) is affected by air pollution. However, how to quantify, evaluate and predict the complex relationship and interactions between ILI infection risk, air pollution and seasonal meteorological factors by using statistical or dynamic models poses a new challenge. Since the beginning of the 21st century, researchers have used statistical or dynamic models to study the relationship between air pollution and non-accidental deaths due to respiratory, cardiovascular and cerebrovascular diseases [10–18]. For example, Tole et al. analyzed the impact of air pollution and meteorological factors on respiratory diseases in Toronto based on a normal linear regression model, and confirmed that there are no reliable statistical data to verify the relationship between air pollution and mortality from respiratory diseases in a specific time [10]. Zhang et al. used a generalized additive model (GAM) to conduct correlation analysis based on air pollutants data (PM10, SO2, NO2) and the number of non-accidental deaths due to respiratory and cardiovascular diseases in Guangzhou, and revealed that non-accidental deaths are affected by haze, age, sex and time lag [6]. Nguyen et al. used a mixed logistic regression model to study the relationship between the numbers of hospitalization days of infected patients with air pollutants (PM10, PM2.5, PM1, SO2, NO, NO2, NOx, CO, O3) in Hanoi, and found that increases of O3 level prolonged the hospital stays [19]. Tang et al. established GAMs and dynamic models for links between the air quality index (AQI) and respiratory infection, and evaluated the impact of air pollution intervention measures on the dynamics of respiratory infection based on the air pollution and ILI data in Xi’an. They found that respiratory infections were affected by the AQI, temperature, humidity and pressure, and identified important intervention measures to reduce air pollution and thus reduce respiratory infections [20].

However, how it remains unclear whether do these meteorological factors and air pollutant affect the spread of ILI epidemics directly or indirectly or not, the question addressed in this study. Therefore, in order to examine the complex relationship between air pollutants, meteorological factors and ILI cases, we will focus on the following issues: (i) Mediation models examining the mediating effects between air pollutants, meteorological factors and ILI cases were applied for the first time; (ii) A discussion of mediating effects of air pollutants on the association between meteorological factors and ILI cases is to explore the optimal mechanism; (iii) The effects of interactions between meteorological factors mediated by air pollutants on the ILI cases are obtained and verified again based on the daily numbers of reported ILI cases by age $0\sim 4$.

The rest of this paper is organized as follows. First of all, we use statistical descriptions for data on the daily numbers of reported ILI cases, air pollutants and meteorological factors for Xi’an city, P.R. China, followed by analyses of these variables using Pearson correlations. Further, six candidate mediation models are established to explore the complex relationships among numbers of ILI cases, air pollutants and meteorological factors. Then the determination coefficient ($R^2$), ordinary least square (OLS) analysis using PROCESS and MEDIATE macros, followed by the Bootstrap method are employed to obtain the optimal model, the best-fit parameter values and significance tests.

## Materials and methods

### Data sources

**Fig. 1:**
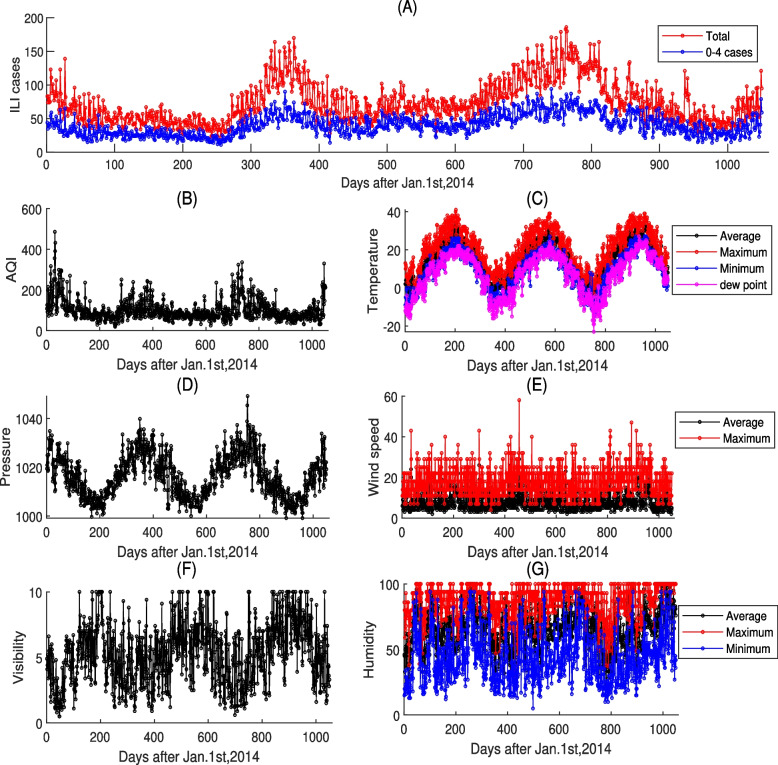
The daily numbers of reported ILI cases, AQI and meteorological factors in Xi’an, Shaanxi Province of China from 1 January 2014 to 15 November 2016

Data on the daily numbers of reported ILI cases data in Xi’an city were collected from the Shaanxi Center for Disease Control and Prevention, including Xi’an Central Hospital, Xi’an No.1 Hospital, Xi’an No.4 Hospital, Xi’an Children’s Hospital, Xi’an No.12 Hospital, Xianyang No.1 Hospital and Xianyang Central Hospital. Data information included the daily numbers of reported ILI cases in clinics and emergency departments (patients who seek medical attention with ILI (defined as with body temperature more than 38°*C*, and cough or sore throat)) for all ages(e.g. 0–4; 5–14; 15–24; 25–59; $\ge$ 60 years). The distribution of ILI cases from 1 January 2014 to 15 November 2016 by total number and age $0\sim 4$ are shown in Fig. 1A. Note that the co-circulation of multiple influenza strains may have slightly influenced the ILI case numbers, particularly after the emergence of a new H3N2 strain in 2015. However, given the stable and consistent reporting efforts during the study period, the data on ILI cases remain relatively accurate. This is further supported by the absence of significant epidemics in Shaanxi Province during this time, with H7N9 outbreaks being confined to southeastern China. Moreover, we assume that each ILI incidence is independent in our dataset. This assumption is reasonable because individuals experiencing repeat attacks are likely to stay in hospitals or seek care at specialized facilities, making the number of repeat ILI cases from sentinel hospitals negligible.

We obtained the data on air pollutants (AQI, PM2.5, PM10, SO2, NO2, CO, O3) from www.tianqihoubao.com (Fig. 1B and Figure A.1 (in the Supporting Information (SI))). The AQI is a quantitative measure used to describe the condition of air quality. Higher AQI values indicate more severe air pollution and greater potential harm to human health. The AQI is typically calculated as a piecewise linear function of the concentrations of various air pollutants (PM2.5, PM10, SO2, NO2, CO, O3) over a specified averaging period [8]. When multiple pollutants are measured at a monitoring site, the highest AQI value among them is reported for that location. Notably, the AQI serves as a tool for Chinese government agencies to inform the public about current air quality conditions and forecasts. Different countries employ their own air quality indices, which correspond to their respective national air quality standards. The AQI is officially categorized into six levels for public alerts and health interventions: excellent, AQI in (0, 50); good, AQI in (51,100); lightly polluted, AQI in (101, 150); moderately polluted, AQI in (151, 200); heavily, AQI in (201, 300); and severely polluted, AQI greater than 300.

Meteorological factors data (daily average temperature, maximum temperature, minimum temperature, dew point, pressure, average wind speed, maximum wind speed, visibility, and average humidity, maximum humidity, minimum humidity) were obtained from the Weather Underground (www.wunderground.com) (Fig. 1C-G). The weather data were measured at the Shaanxi Meteorological Office at the International Airport of Xianyang, adjoining Xi’an. This station provides representative data that are consistent with International World Meteorological Organization standards.

### Mediation analysis

**Fig. 2:**
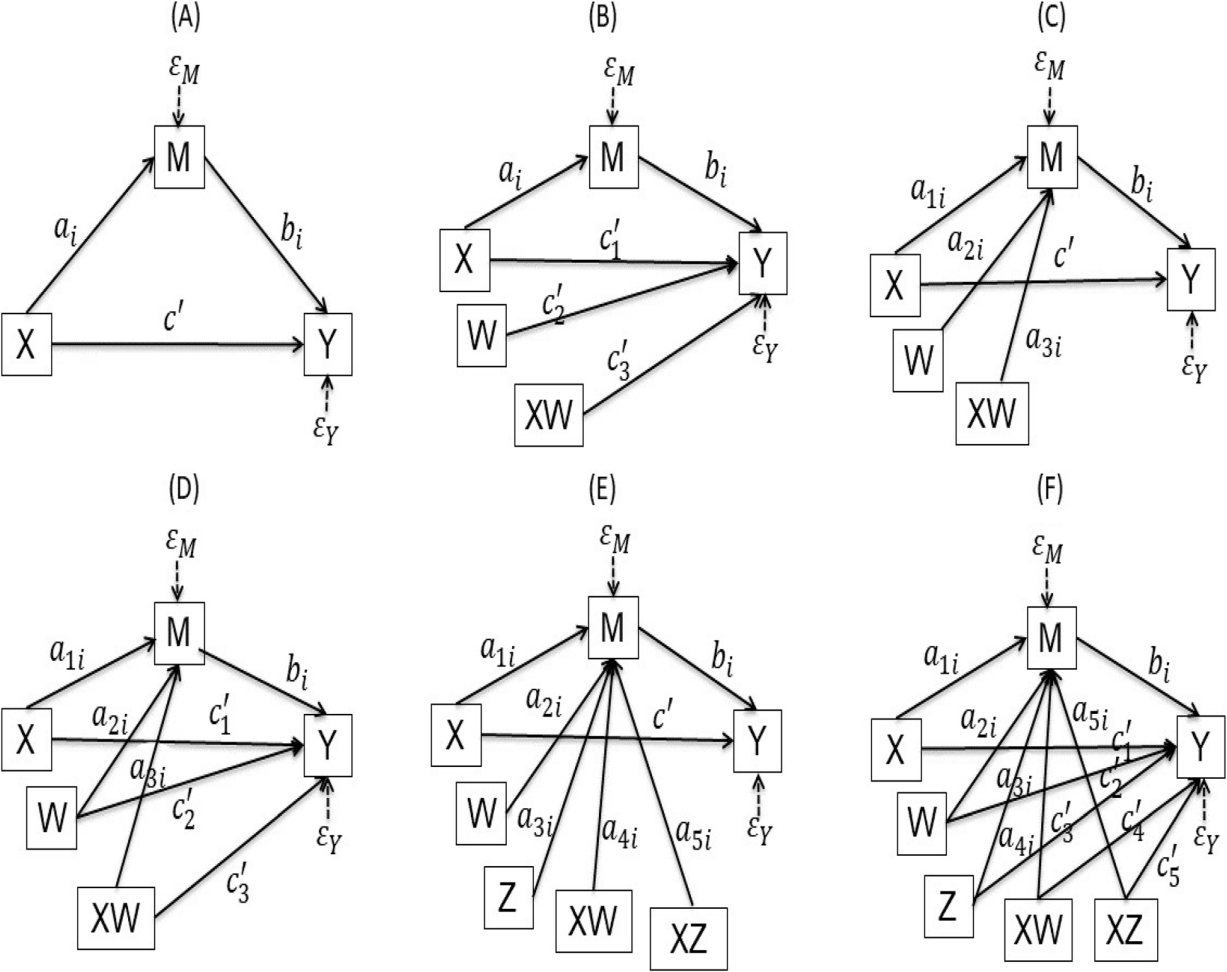
Statistical diagrams of the mediation models. For explanations of symbols see text

Mediation analysis is used to evaluate evidence from studies designed to test hypotheses about how some causal antecedent variable *X* transmits its effect on a consequent variable *Y* [21–28]. So, to explore the direct impact of meteorological factors on the ILI epidemic and the mediating effect of air pollution on the association between meteorological factors and ILI epidemic, we employed simple mediation models with one mediator variable and moderated mediation models with one mediator variable, which are shown in statistical diagrams in Fig. 2 and conceptual diagrams in Figure B.1 of the SI.

#### The simple statistical diagram and its mediation model

The simple mediation model (Fig. 2A) contains two consequent variables (air pollutants (*M*) and ILI cases (*Y*)) and two antecedent variables (meteorological factors (*X*) and air pollutants (*M*)), with *X* causally influencing *Y* and *M*, and *M* causally influencing *Y*.

Therefore, there are two pathways by which *X* can influence *Y*. One pathway is the direct effect of *X* on *Y*, which passes from *X* to *Y* without passing through *M*. The other pathway is the indirect effect of *X* on *Y*, which represents how *Y* is influenced by *X* through a causal sequence in which *X* influences *M*, which in turn influences *Y*. Thus, the statistical diagram represents the following equations:

1 $$\begin{aligned} M & = i_M + a_i X + \varepsilon _M,\nonumber \\ Y & = i_Y + c^{\prime } X + b_i M + \varepsilon _Y, \end{aligned}$$

where $i_M$, $i_Y$ are constant parameters; $\varepsilon _M$, $\varepsilon _Y$ are error terms; $a_i$, $b_i$ and $c^{\prime }$ are regression coefficients. These coefficients could depict the causal influences of antecedent variables on the consequent variables, and could be estimated by ordinary least square (OLS) methods using the PROCESS and MEDIATE macros in SPSS [24, 28]. The detailed explanation of these coefficients are shown in Appendix B of SI.

#### The statistical diagrams and their moderated mediation models

Sometimes, a variable’s effects cannot be quantified with a single number, such as the effect of *X* on *Y* is moderated by *W* (Fig. 2B), or the effect of *X* on *M* is moderated by *W* (Fig. 2C), or both the effect of *X* on *Y* and the effect of *X* on *M* are moderated by *W* (Fig. 2D), or the effect of *X* on *Y* is moderated by *W* and *Z* (Fig. 2E), or both the effect of *X* on *Y* and the effect of *X* on *M* are moderated by *W* and *Z* (Fig. 2F). Here the moderated variables *W* and *Z* are meteorological factors [24, 28].

For Fig. 2B, we assume linear moderation of the direct effect of *X* by *W*, the two equations are

2 $$\begin{aligned} M & = i_M + a_i X + \varepsilon _M,\nonumber \\ Y & = i_Y + c_1^{\prime } X + c_2^{\prime } W + c_3^{\prime } XW + b_i M + \varepsilon _Y. \end{aligned}$$

The *X* on *Y* moderated by *W* is a conditional direct effect, which is $c_1^{\prime } + c_3^{\prime } W$; the indirect effect of *X* on *Y* through *M* is $a_i \times b_i$. The interpretation of parameters in model (2) is similar to model (1).

The equations of Fig. 2C are

3 $$\begin{aligned} M & = i_M + a_{1i} X + a_{2i} W + a_{3i} XW + \varepsilon _M,\nonumber \\ Y & = i_Y + c^{\prime } X + b_i M + \varepsilon _Y. \end{aligned}$$

The direct effect of *X* on *Y* is $c^{\prime }$, the conditional indirect effect *X* on *Y* through *M* moderated by *W* is $(a_{1i} + a_{3i} W) b_i$.

The equations of Fig. 2D are

4 $$\begin{aligned} M & = i_M + a_{1i} X + a_{2i} W + a_{3i} XW + \varepsilon _M,\nonumber \\ Y & = i_Y + c_1^{\prime } X + c_2^{\prime } W + c_3^{\prime } XW + b_i M + \varepsilon _Y. \end{aligned}$$

The conditional direct effect of *X* on *Y* moderated by *W* is $c_1^{\prime } + c_3^{\prime } W$, the conditional indirect effect of *X* on *Y* through *M* moderated by *W* is $(a_{1i} + a_{3i} W) b_i$.

The equations of Fig. 2E are

5 $$\begin{aligned} M & = i_M + a_{1i} X + a_{2i} W + a_{3i} Z + a_{4i} XW + a_{5i} XZ + \varepsilon _M,\nonumber \\ Y & = i_Y + c^{\prime } X + b_i M + \varepsilon _Y. \end{aligned}$$

The direct effect of *X* on *Y* is $c^{\prime }$, the conditional indirect effect *X* on *Y* through *M* moderated by *W* and *Z* is $(a_{1i} + a_{4i} W + a_{5i}Z) b_i$.

The equations of Fig. 2F are

6 $$\begin{aligned} M & = i_M + a_{1i} X + a_{2i} W + a_{3i} Z + a_{4i} XW + a_{5i} XZ + \varepsilon _M,\nonumber \\ Y & = i_Y + c_1^{\prime } X + c_2^{\prime } W + c_3^{\prime } Z + c_4^{\prime } XW + c_5^{\prime } XZ + b_i M + \varepsilon _Y. \end{aligned}$$

The conditional direct effect of *X* on *Y* moderated by *W* and *Z* is $c_1^{\prime } + c_4^{\prime } W + c_5^{\prime } Z$, the conditional indirect effect *X* on *Y* through *M* moderated by *W* and *Z* is $(a_{1i} + a_{4i} W + a_{5i}Z) b_i$.

#### Statistical inference and model selection by mediation analysis

Inference about the direct effect and total effect of *X* on *Y* for model (1) is carried out by OLS a regression program in the PROCESS procedure, the confidence intervals $c^{\prime }$ and *c* can be constructed [28]. The detailed processes of the statistical inference are shown in the Appendix B of the SI. The bootstrap method is employed to infer about the indirect effect *X* on *Y* through *M* for model (1), detailed in the Appendix B of the SI. Besides, inference about a direct effect (conditional direct effect) and an indirect effect (conditional indirect effect) of moderated mediation models (2-6) is similar to model (1) calculated by bootstrap method [28]. Next, the coefficient of determination, $R^2$, is a key indicator for evaluating the goodness of model fitting. It reflects the degree of consistency between the estimated values, $\hat{y}_i$, and the observed values, $y_i$, or, in other words, the proportion of the variance in the data that the model can explain [28]. Mathematically, $R^2$ is defined as:

$$\begin{aligned} R^2 = 1 - \frac{\sum (y_i-\hat{y}_i)^{2}}{\sum (y_i-\bar{y}_i)^{2}} \end{aligned}$$

where $\bar{y}_i$ is the mean of all observed values. The value of $R^2$ ranges from 0 to 1. The closer the value is to 1, the better the model’s fit and vice versa.

## Results

### Distribution characteristics of ILI cases, air pollutants and meteorological factors

Distributions of the data on daily numbers of reported ILI cases, air pollutants and meteorological factors in Xi’an, Shaanxi province of China, from 1 January 2014 to 15 November 2016 are depicted in Fig. 1, Figure A.1 and Table 1. During the study period, the trend of the daily numbers of reported ILI cases show periodic variations with peaks in the winter season (Fig. 1A). The mean daily numbers of all ILI cases was 73.93, most of which (40.53) were in children of age $0\sim 4$. This proportion exceeded 50% of all cases. Similarly, the air pollutants (AQI, PM2.5, PM10, SO2, NO2, CO, O3) behave periodically, peaking in the winter, as do the meteorological factors (daily average temperature, maximum temperature, minimum temperature, dew point, pressure, visibility) but they were lowest in the winter. The median (25th percentile −75th percentile) daily average temperature, average humidity, and pressure were 16°C(6°C-23°C), 67%(56%−79%), and 1015.75 Pa(1008.95Pa-1022.75Pa), reflecting the climate of Xi’an city which varies between being semi-arid and humid.

**Table 1:** Statistical descriptions of the daily numbers of reported ILI cases, air pollutants and meteorological data in Xi’an, Shaanxi province of China from 1 January 2014 to 15 November 2016

Variables	Min	Q1	Median	Q3	Max	Mean $\varvec{\pm }$ SD
Daily number of reported ILI cases						
Total	22	50.00	66.00	90.00	186	73.93 ± 31.176
Age 0 $\sim$4 cases	11	28.00	38.00	50.25	94	40.53 ± 15.737
Air Pollutant						
AQI	19	69.00	87.00	114.00	486	102.17 ± 54.546
PM2.5($\mu g/m^3$)	11	34.00	50.00	74.25	527	63.60 ± 48.528
PM10 ($\mu g/m^3$)	17	80.00	111.00	165.00	659	133.00 ± 77.532
SO2($\mu g/m^3$)	2	10.00	17.00	31.00	145	23.84 ± 20.890
NO2($\mu g/m^3$)	13	33.00	42.00	54.00	101	44.56 ± 15.848
CO ($mg/m^3$)	0.61	1.1600	1.5200	2.0325	5.37	1.7133 ± 0.75053
O3 ($\mu g/m^3$)	5	21.00	36.00	62.00	141	43.07 ± 26.787
Meteorological data						
Average temperature(°C)	−8	6.00	16.00	23.00	34	14.88 ± 9.868
Maximum temperature (°C)	−3	12.00	22.00	29.00	41	20.53 ± 10.272
Minimum temperature(°C)	−17	1.00	10.00	18.00	27	9.14 ± 10.045
Average humidity(%)	21	56.00	67.00	79.00	100	66.49 ± 15.816
Maximum humidity(%)	26	81.00	93.00	100.00	100	87.94 ± 12.281
Minimum humidity(%)	5	29.00	40.00	54.00	94	43.46 ± 19.197
Pressure(Pa)	999.17	1008.95	1015.75	1022.75	1049.17	1016.2931 ± 8.79841
Dew point(°C)	−23	−1.00	10.00	16.00	27	7.39 ± 10.508
Wind speed(Km/h)	2	5.00	7.00	10.00	30	7.81 ± 4.035
Maximum wind speed(Km/h)	4	14.00	18.00	22.00	58	17.90 ± 7.018
Visibility(Km)	0.5	3.400	5.200	6.900	10.0	5.277 ± 2.3082

*Min *Minimum value, *Q1 *25th percentile, *Q3 *75th percentile, *Max *Maximum value

### Pearson correlations between numbers of ILI cases, air pollutants and meteorological factors

In what follows, $Y_1$ and $Y_2$ denote the total number of daily reported ILI cases and the number of daily reported ILI cases by age $0\sim 4$, respectively. We use $Z_1$ for AQI, $Z_2$ for PM2.5, $Z_3$ for PM10, $Z_4$ for SO2, $Z_5$ for NO2, $Z_6$ for CO, $Z_7$ for O3, $X_1$ for average temperature, $X_2$ for maximum temperature, $X_3$ for minimum temperature, $X_4$ for average humidity, $X_5$ for maximum humidity, $X_6$ for minimum humidity, $X_7$ for pressure, $X_8$ for dew point, $X_9$ for wind speed, $X_{10}$ for maximum wind speed, $X_{11}$ for visibility, respectively. The objective of the data analysis was to quantify the association among ILI cases, air pollutants, and meteorological factors. We adopt the Pearson correlation method [29, 30] to explore the association between them during the specified period. Some results are summarized in Table 2.

**Table 2:** Pearson correlation analysis between data on the daily numbers of reported ILI cases, air pollutants and meteorological factors in Xi’an, Shaanxi province of China from 1 January 2014 to 15 November 2016

	$\varvec{Y}_{\varvec{1}}$	$\varvec{Y}_{\varvec{2}}$	$\varvec{Z}_{\varvec{1}}$	$\varvec{Z}_{\varvec{2}}$	$\varvec{Z}_{\varvec{3}}$	$\varvec{Z}_{\varvec{4}}$	$\varvec{Z}_{\varvec{5}}$	$\varvec{Z}_{\varvec{6}}$	$\varvec{Z}_{\varvec{7}}$	$\varvec{X}_{\varvec{1}}$	$\varvec{X}_{\varvec{2}}$	$\varvec{X}_{\varvec{3}}$	$\varvec{X}_{\varvec{4}}$	$\varvec{X}_{\varvec{5}}$	$\varvec{X}_{\varvec{6}}$	$\varvec{X}_{\varvec{7}}$	$\varvec{X}_{\varvec{8}}$	$\varvec{X}_{\varvec{9}}$	$\varvec{X}_{\varvec{10}}$	$\varvec{X}_{\varvec{11}}$
$Y_1$	1																			
$Y_2$	0.93**	1																		
$Z_1$	0.24**	0.20**	1																	
$Z_2$	0.23**	0.17**	0.95**	1																
$Z_3$	0.28**	0.24**	0.97**	0.90**	1															
$Z_4$	0.38**	0.26**	0.68**	0.71**	0.66**	1														
$Z_5$	0.28**	0.23**	0.66**	0.66**	0.69	0.59**	1													
$Z_6$	0.40**	0.32**	0.72**	0.76**	0.73**	0.78**	0.64**	1												
$Z_7$	−0.33**	−0.21**	−0.34*	−0.38**	−0.39**	−0.55**	−0.36**	−0.60**	1											
$X_1$	−0.58**	−0.47**	−0.43*	−0.46**	−0.45**	−0.72**	−0.38**	−0.71**	0.78**	1										
$X_2$	−0.53**	−0.42**	−0.39**	−0.44**	−0.40**	−0.66**	−0.28**	−0.65**	0.80**	0.97**	1									
$X_3$	−0.59**	−0.48**	−0.45**	−0.45**	−0.47**	−0.73**	−0.45**	−0.72**	0.71**	0.96**	0.87**	1								
$X_4$	−0.24**	−0.23**	−0.05	0.08**	−0.12**	−0.21**	−0.07**	−0.02	−0.23**	0.07**	−0.08**	0.21**	1							
$X_5$	−0.23**	−0.20**	−0.08**	0.00	−0.15**	−0.23**	−0.01	−0.07**	−0.09**	0.13**	0.07*	0.17**	0.81**	1						
$X_6$	−0.22**	−0.21**	−0.08*	0.05	−0.14**	−0.20**	−0.19**	−0.06*	−0.21**	0.06	−0.14**	0.25*	0.89**	0.55**	1					
$X_7$	0.50**	0.41**	0.25**	0.27**	0.27**	0.51**	0.24**	0.47**	−0.70**	−0.86**	−0.86**	−0.81**	−0.04	−0.08**	−0.04	1				
$X_8$	−0.61**	−0.51**	−0.41**	−0.39**	−0.45**	−0.72**	−0.37**	−0.65**	0.63**	0.92**	0.84**	0.95**	0.43**	0.41**	0.39**	−0.79**	1			
$X_9$	−0.09**	−0.06**	−0.22**	−0.22**	−0.22**	−0.23**	−0.48**	−0.27**	0.29**	0.19**	0.11**	0.27**	−0.14**	−0.26**	0.11**	−0.16**	0.15**	1		
$X_{10}$	−0.10**	−0.07*	−0.21**	−0.23**	−0.20**	−0.22**	−0.40**	−0.27**	0.28**	0.21**	0.15**	0.25**	−0.14**	−0.22**	0.04	−0.20**	0.15**	0.82**	1	
$X_{11}$	−0.16**	−0.10**	−0.53**	−0.62**	−0.50**	−0.43**	−0.37**	−0.59**	0.54**	0.47**	0.52**	0.38**	−0.47**	−0.29**	−0.44**	−0.35**	0.25**	0.19**	0.24**	1

Significance of correlation coefficient different from zero: ** represents $p<0.01$, * represents $p<0.05$. $Y_1$ and $Y_2$ denote the total number of daily reported ILI cases and the number of daily reported ILI cases by age $0\sim 4$, respectively. We use $Z_1$ for AQI, $Z_2$ for PM2.5, $Z_3$ for PM10, $Z_4$ for SO2, $Z_5$ for NO2, $Z_6$ for CO, $Z_7$ for O3, $X_1$ for average temperature, $X_2$ for maximum temperature, $X_3$ for minimum temperature, $X_4$ for average humidity, $X_5$ for maximum humidity, $X_6$ for minimum humidity, $X_7$ for pressure, $X_8$ for dew point, $X_9$ for wind speed, $X_{10}$ for maximum wind speed, $X_{11}$ for visibility, respectively

We concluded that the AQI ($Z_1$), PM2.5($Z_2$), PM10($Z_3$), SO2($Z_4$), NO2($Z_5$), CO($Z_6$) and pressure ($X_7$) are statistically significantly positive correlated with the total numbers of daily reported ILI cases ($Y_1$) and the numbers of daily reported ILI cases by age $0\sim 4$ ($Y_2$) over the study period, and meanwhile, the other variables are statistically significantly negatively correlated with $Y_1$ and $Y_2$.

Among these correlations, those between average temperature ($X_1$), maximum temperature ($X_2$), minimum temperature ($X_3$), pressure ($X_7$), dew point ($X_8$) and the total numbers of daily reported ILI cases ($Y_1$), the numbers of daily reported ILI cases by age ($Y_2$) are almost equivalent (approximately 0.5), as were those for average humidity ($X_4$), maximum humidity ($X_5$), visibility ($X_{11}$)(approximately 0.2) and wind speed ($X_9$), maximum wind speed ($X_{10}$)(approximately 0.1). We also reported in Table 2 the correlations between each pair of the other variables. This report showed that some of these variables have statistically significant correlations, and they are highly correlated or moderately correlated according to the labelling systems roughly categorized (low or weak correlations ($|\gamma |\le 0.35$), moderate correlations ($0.36\le |\gamma |\le 0.67$) and strong/high correlations ($0.68\le |\gamma |\le 1.0$) [30]). In particular, we noticed that PM2.5 ($Z_2$), PM10 ($Z_3$), SO2 ($Z_4$), CO($Z_6$) are strongly correlated to AQI ($Z_1$) (NO2 ($Z_5$)(moderately correlated), O3 ($Z_7$)(weakly correlated)), average temperature ($X_1$), maximum temperature ($X_2$), dew point ($X_8$) are strongly correlated to minimum temperature ($X_3$), and average humidity ($X_4$) (minimum humidity ($X_6$)) is strongly(moderately) correlated to maximum humidity ($X_5$), wind speed ($X_9$) is strongly correlated to maximum wind speed ($X_{10}$), and the number of daily reported ILI cases by age $0\sim 4$ ($Y_2$) is strongly correlated to the total number of daily reported ILI cases ($Y_1$). See Table 2 for details. Therefore, in the following sections, we firstly study the association between AQI ($Z_1$), the minimum temperature ($X_3$), the maximum humidity ($X_5$), pressure ($X_7$), maximum wind speed($X_{10}$) and ILI cases ($Y_1$).

### Main results of mediation analysis

#### Model selection for the mediation model and moderated mediation models

All variables have been centralized. From Table 3, we can obtain that when the variable $X = X_3$, the determination coefficient $R^2$ of the model (6)($X = X_3$, $W = X_5$, $Z = X_7$) is the highest (0.2917 and 0.3846), by comparing $R^2$ in the model (1-6), and the model test statistics are significant. That is, the model (6) could better reveal the mediating effect of AQI on the association between minimum temperature, maximum humidity, pressure and the total numbers of daily reported ILI cases. Besides, the conditional direct effect of minimum temperature on the total numbers of daily reported ILI cases is statistically significant, while the conditional indirect effect of minimum temperature on the total numbers of daily reported ILI cases through AQI is not statistically significant. The mediating effect of the moderated mediation variables maximum humidity($W = X_5$) and pressure ($Z = X_7$) are not significant with minimum temperature ($X = X_3$).

**Table 3:** The mediating effects for models (1-6) ($Y=Y_1$, $M=AQI$)

	$\varvec{R}^{\varvec{2}}\!\varvec{(X \rightarrow M)}$ (Yes/No)	$\varvec{R}^{\varvec{2}}\!\varvec{(M\rightarrow Y)}$ (Yes/No)	$\varvec{R}^{\varvec{2}}\!\varvec{(X\rightarrow Y)}$ (Yes/No)	Direct effect (Conditional direct effects) (Yes/No)	Indirect effect (Conditional indirect effects)(Yes/No)	Total effect (Yes/No)	Index of moderated mediation (Yes/No)
model (1) ($X=X_3$)	0.1991(Yes)	0.3474(Yes)	0.3470(Yes)	Yes	No	Yes	/
model (1) ($X=X_5$)	0.0069(Yes)	0.1017(Yes)	0.0509(Yes)	Yes	Yes	Yes	/
model (1) ($X=X_7$)	0.0622(Yes)	0.2616(Yes)	0.2464(Yes)	Yes	Yes	Yes	/
model (1) ($X=X_{10}$)	0.0459(Yes)	0.0614(Yes)	0.0096(Yes)	No	Yes	Yes	/
model (2) ($X=X_3$, $W=X_5$)	0.1991(Yes)	0.3634(Yes)	/	Yes	No	/	/
model (2) ($X=X_3$, $W=X_7$)	0.1991(Yes)	0.3764(Yes)	/	Yes	No	/	/
model (2) ($X=X_3$, $W=X_{10}$)	0.1991(Yes)	0.3535(Yes)	/	Yes	No	/	/
model (2) ($X=X_5$, $W=X_3$)	0.0069(Yes)	0.3634(Yes)	/	Yes	No	/	/
model (2) ($X=X_5$, $W=X_7$)	0.0069(Yes)	0.2947(Yes)	/	Yes	Yes	/	/
model (2) ($X=X_5$, $W=X_{10}$)	0.0069(Yes)	0.1190(Yes)	/	Yes	Yes	/	/
model (3) ($X=X_3$, $W=X_5$)	0.2471(Yes)	0.3474(Yes)	/	Yes	No	/	No
model (3) ($X=X_3$, $W=X_7$)	0.2493(Yes)	0.3474(Yes)	/	Yes	No	/	No
model (3) ($X=X_3$, $W=X_{10}$)	0.2324(Yes)	0.3474(Yes)	/	Yes	No	/	No
model (3) ($X=X_5$, $W=X_3$)	0.2471(Yes)	0.1017(Yes)	/	Yes	Yes	/	Yes
model (3) ($X=X_5$, $W=X_7$)	0.0907(Yes)	0.1017(Yes)	/	Yes	Yes/No	/	Yes
model (3) ($X=X_5$, $W=X_{10}$)	0.0648(Yes)	0.1017(Yes)	/	Yes	Yes/No	/	No
model (4) ($X=X_3$, $W=X_5$)	0.2471(Yes)	0.3634(Yes)	/	Yes	No	/	No
model (4) ($X=X_3$, $W=X_7$)	0.2493(Yes)	0.3764(Yes)	/	Yes	No	/	No
model (4) ($X=X_3$, $W=X_{10}$)	0.2324(Yes)	0.3535(Yes)	/	Yes	No	/	No
model (4) ($X=X_5$, $W=X_3$)	0.2471(Yes)	0.3634(Yes)	/	Yes	No	/	No
model (4) ($X=X_5$, $W=X_7$)	0.0907(Yes)	0.2947(Yes)	/	Yes	Yes/No	/	Yes
model (4) ($X=X_5$, $W=X_{10}$)	0.0648(Yes)	0.1190(Yes)	/	Yes	Yes/No	/	No
model (5) ($X=X_3$, $W=X_5$, $Z=X_7$)	0.2917(Yes)	0.3474(Yes)	/	Yes	No	/	W(No),Z(No)
model (5) ($X=X_3$, $W=X_{10}$, $Z=X_7$)	0.2868(Yes)	0.3474(Yes)	/	Yes	No	/	W(No),Z(No)
model (5) ($X=X_5$, $W=X_3$, $Z=X_7$)	0.2779 (Yes)	0.1017(Yes)	/	Yes	Yes/No	/	W(Yes),Z(No)
model (5) ($X=X_5$, $W=X_{10}$, $Z=X_7$)	0.1262(Yes)	0.1017(Yes)	/	Yes	No	/	W(No),Z(Yes)
**model (**6**)** ($X=X_3$, $W=X_5$, $Z=X_7$)	**0.2917(Yes)**	**0.3846(Yes)**	/	**Yes**	**No**	/	**W(No),Z(No)**
model (6) ($X=X_3$, $W=X_{10}$, $Z=X_7$)	0.2868(Yes)	0.3474(Yes)	/	Yes	No	/	W(No),Z(No)
**model (**6**)** ($X=X_5$, $W=X_3$, $Z=X_7$)	**0.2779 (Yes)**	**0.3656(Yes)**	/	**Yes/No**	**No**	/	**W(No),Z(No)**
model (6) ($X=X_5$, $W=X_{10}$, $Z=X_7$)	0.1262(Yes)	0.3000(Yes)	/	Yes	Yes/No	/	W(No),Z(Yes)

Yes/No in brackets indicates whether the indicator is statistically significant or not. $Y_1$ denote the total number of daily reported ILI cases. We use $X_3$ for minimum temperature, $X_5$ for maximum humidity, $X_7$ for pressure, $X_{10}$ for maximum wind speed, respectively

In addition, when the variable $X=X_5$, $R^2$ of the model (6) ($X = X_5$, $W = X_3$, $Z = X_7$) is highest (0.2779 and 0.3656), by comparing $R^2$ in the model (1-6), and the model test statistics are significant. That is, the model (6) could better reveal the mediating effect of AQI on the association between maximum humidity, minimum temperature, pressure and the total numbers of daily reported ILI cases. Besides, the conditional direct effect of maximum humidity on the total numbers of daily reported ILI cases is statistically significant in most cases, while the conditional indirect effect of maximum humidity on the total numbers of daily reported ILI cases through it is not statistically significant. The mediating effect of the moderated mediation variables both visibility ($W = X_{11}$) and pressure ($Z = X_7$) are not significant with maximum humidity ($X = X_5$).

#### The results of mediation analysis

**Fig. 3:**
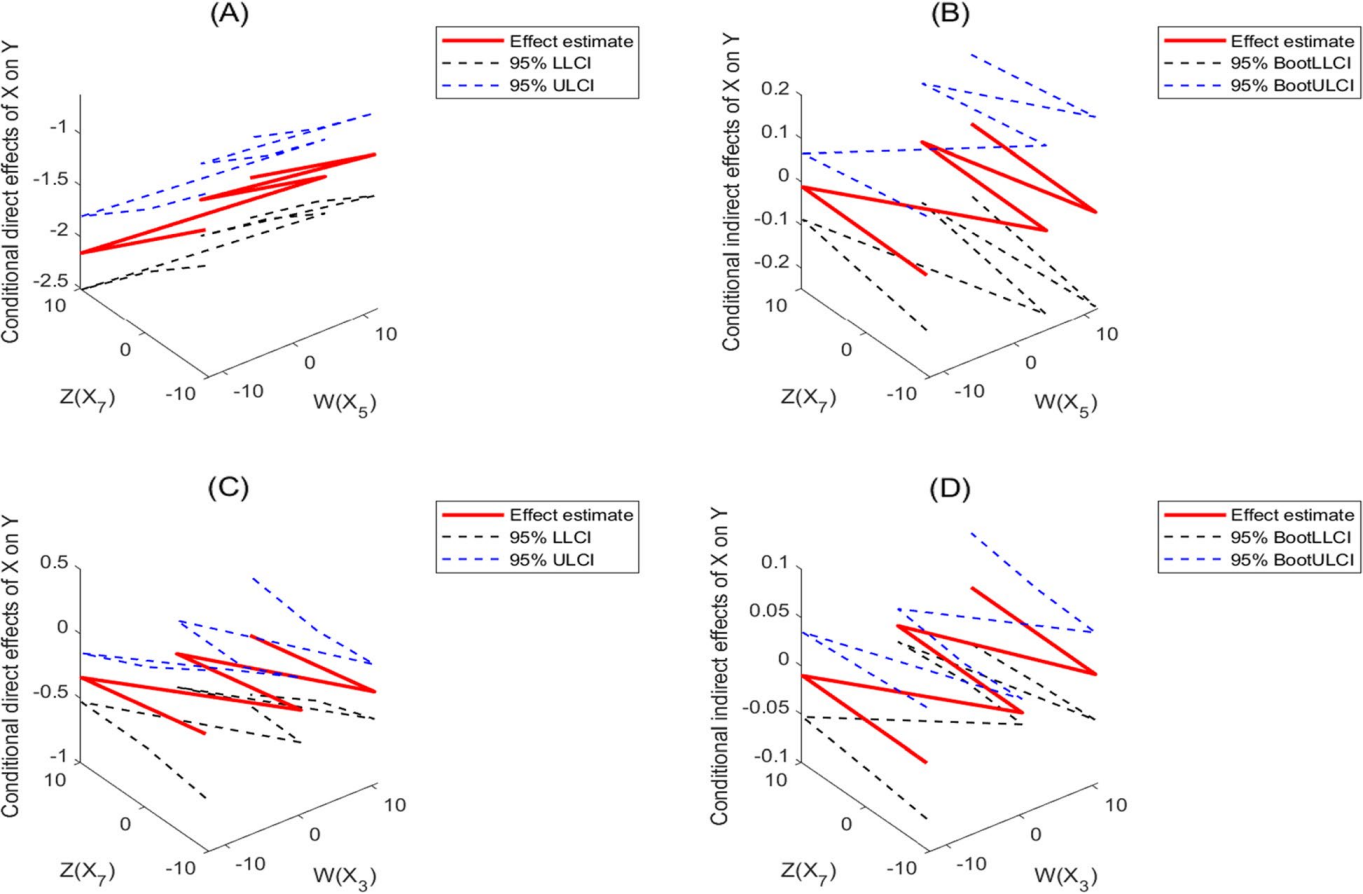
Conditional direct and indirect effects of X on Y. **A** and **B** For model (6) ($X=X_3$, $W=X_5$, $Z=X_7$); **C** and **D** For model (6) ($X=X_5$, $W=X_3$, $Z=X_7$). The effect estimate is estimation of conditional direct and indirect effects based on Model (6), which are obtained based on the estimated formulas $-1.5822 + 0.0029 X_5 - 0.0550 X_7$, $- 0.0195 - 0.0005 X_5 + 0.0004 X_7$, $- 0.3267 - 0.0087 X_3 - 0.0116 X_7$, $0.0008 + 0.0009 X_3 + 0.0001 X_7$ in the main text, respectively. The LLCI and ULCI are lower limit confidence interval and upper limit confidence interval, respectively. The BootLLCI and BootULCI are bootstrap lower limit confidence interval and bootstrap upper limit confidence interval, respectively. The detailed are shown in Appendix B of SI. W and Z values in conditional figures are the 16th, 50th, and 84th percentiles

Table 4 and Fig. 3 shown the results of our mediation analysis. For model (6) ($X = X_3$, $W = X_5$, $Z = X_7$), AQI partially mediated the association of minimum temperature and pressure with the total numbers of daily reported ILI cases, but which is not significant(interval estimate for conditional direct effect include zero). The conditional direct effect of minimum temperature on ILI infections moderated by maximum humidity and pressure is $- 1.5822 + 0.0029 X_5 - 0.0550 X_7$, and which is significant. The conditional direct effect of minimum temperature indicated that 1 °C increase in minimum temperature was associated with a 1.5822 (95% CI: 1.2933, 1.8710) decrease in daily numbers of ILI cases. Besides, the conditional indirect effect of minimum temperature on ILI infections through AQI moderated by maximum humidity and pressure is $-0.0195 - 0.0005 X_5 + 0.0004 X_7$, which is not significant.

**Table 4:** Mediating effects of AQI on the association between meteorological factors and the total numbers of daily reported ILI cases for model (6)

		Model (6) ($\varvec{X=X}_{\varvec{3}}$,$\varvec{W=X}_{\varvec{5}}$, $\varvec{Z=X}_{\varvec{7}}$)		model (6) ($\varvec{X=X}_{\varvec{5}}$,$\varvec{W=X}_{\varvec{3}}$,$\varvec{Z=X}_{\varvec{7}}$)	
		M	Y	M	Y
	Variables	AQI($\varvec{Z}_{\varvec{1}}$)	ILI new cases($\varvec{Y}_{\varvec{1}}$)	AQI($\varvec{Z}_{\varvec{1}}$)	ILI new cases($\varvec{Y}_{\varvec{1}}$)
	Constant	7.8476**(4.0655, 11.6297)	−4.0010**(−6.0329, −1.9690)	2.1286(−0.7544, 5.0115)	0.0813(−1.4654, 1.6280)
	TempMin ($X_3$)	−3.9840**(−4.4689, −3.4990)	−1.5822**(−1.8710, −1.2933)		
X	HumiMax ($X_5$)			−0.0925(−0.3316, .1467)	−0.3267**(−0.4550, −0.1985)
	HumiMax ($X_5$)	−0.2457*(−0.4889, −0.0024)	−0.2347**(−0.3646, −0.1048)		
W	TempMin ($X_3$)			−4.0545**(−4.5460, −3.5629)	−1.6121**(−1.9067, −1.3174)
Z	Pressure ($X_7$)	−1.6632**(−2.2179, −1.1085)	0.1332(−0.1673, 0.4337)	−1.8915**(−2.4481, −1.3348)	0.2318(−0.0728, 0.5364)
XW	TempMin $\times$ HumiMax ($X_3$ $\times$ $X_5$)	−0.0970**(−0.1211, −0.0728)	0.0029(−0.0103, 0.0162)	−0.1057**(−0.1481, −0.0634)	−0.0087 (−0.0316, 0.0143)
XZ	TempMin $\times$ Pressure ($X_3$ $\times$ $X_7$)	0.0808(0.0461, 0.1156)	−0.0550**(−0.0737, −0.0362)		
	HumiMax $\times$ Pressure ($X_5$ $\times$ $X_7$)			−0.0124(−0.0551, 0.0303)	−0.0116(−0.0345, 0.0113)
M	AQI ($Z_1$)		0.0049(−0.0275, 0.0372)		−0.0087(−0.0413, 0.0238)
		$R^2=0.2917$**	$R^2=0.3846$**	$R^2=0.2779$**	$R^2=0.3656$**

This table reports the model (6) estimated coefficients and 95% confidence intervals of interest variable and mediator. Significance of coefficient different from zero: ** represents $p<0.01$, * represents $p<0.05$. $Y_1$ denote the total number of daily reported ILI cases. We use $Z_1$ for AQI, $X_3$ for minimum temperature, $X_5$ for maximum humidity, $X_7$ for pressure, $X_{10}$ for maximum wind speed, respectively

For model (6)($X = X_5$, $W = X_3$, $Z = X_7$), AQI partially mediated the association of minimum temperature and pressure with the total numbers of daily reported ILI cases, but which is not significant. The conditional direct effect of maximum humidity on ILI infections moderated by minimum temperature and pressure is $-0.3267 -0.0087 X_3 -0.0116 X_7$, which is statistically significant in most cases. The conditional direct effect showed that 1% increase in maximum humidity was associated with a 0.3267 (95% CI: 0.1985 to 0.4550) decrease in daily ILI cases. Besides, the conditional indirect effect of maximum humidity on ILI infections through AQI moderated by minimum temperature and pressure is $0.0008 + 0.0009 X_3 + 0.0001 X_7$, which is significant in most cases.

In addition, the mediating effects of other air pollutants (PM2.5, PM10, SO2, NO2, CO, O3) on the association between meteorological factors and the total numbers of daily reported ILI cases for model (6) ($Y=Y_1$) are shown in Table B.1. From Table B.1, firstly, we can obtain that the mediating effect of other air pollutants (PM2.5, PM10, SO2, NO2, CO, O3) on the association between minimum temperature, maximum humidity, pressure and the total numbers of daily reported ILI cases. The conditional direct effect of minimum temperature on the total numbers of daily reported ILI cases is statistically significant, while the conditional indirect effect of minimum temperature on the total numbers of daily reported ILI cases through air pollutants (SO2, NO2, O3) is statistically significant. The mediating effect of the moderated mediation variables maximum humidity and pressure is inconsistent statistically with minimum temperature through air pollutants.

Next, the mediating effect of other air pollutants (PM2.5, PM10, SO2, NO2, CO, O3) on the association between maximum humidity, minimum temperature,pressure and the total numbers of daily reported ILI cases is also shown in Table B.1. The conditional direct effect of maximum humidity on the total numbers of daily reported ILI cases is sometimes statistically significant, and also the conditional indirect effect of maximum humidity on the total numbers of daily reported ILI cases through air pollutants (SO2, O3) is sometimes statistically significant. The mediating effect of the moderated mediation variables minimum temperature and pressure is statistically with maximum humidity through air pollutants(SO2, O3).

Lastly, we analysed the mediating effects of AQI on the association between meteorological factors and the daily numbers of reported ILI cases by age $0\sim 4$ ($Y=Y_2$, $M=AQI$) for model (1-6), as shown in Table B.2. Table B.2 shown that the mediating effect of AQI on the association between minimum temperature, maximum humidity, pressure and the daily reported ILI cases by age $0\sim 4$, which was almost the same as that of the total numbers of daily reported ILI cases. Besides, the results for the mediating effect of AQI on the association between maximum humidity, minimum temperature, pressure and the daily numbers of reported ILI cases by age $0\sim 4$, were also almost same.

## Discussion

The purpose of this paper was to determine the underlying mechanism of the association between meteorological factors and the spread of ILI by assessing the mediating effect of air pollutants, an approach which differs from those in previous studies [8, 10–14, 14–16, 16–18, 31], which aimed to examine the relationship between air pollutants and ILI infections. In this study, we found that the AQI, PM2.5, PM10, SO2, NO2, CO and pressure are statistically significantly positive correlated with ILI cases, while the other meteorological factors are statistically significantly negatively correlated with ILI cases by Pearson correlation analyses. The mediation analysis showed that AQI did not significantly mediated the association between maximum humidity, minimum temperature, pressure and ILI infections. These results provide evidence that there may be many factors affecting ILI outbreaks, and the means by which meteorological factors and air pollutants affect ILI epidemics is very complex.

Some of our results are consistent with previous studies. Firstly, for the positive relationship between air pollutants and ILI cases, Carugno et al. found that the mortality of non-accidental deaths were affected by PM10 and NO2 concentration, season and age [11]. Besides, Hao et al. reported that exposure to air pollutants SO2 and NO2 would significantly increase the incidence rate of mumps [32]. Also, Luong et al. showed that the increase in PM2.5 concentration would lead to an increase in the number of children infected with ALRI virus [33]. In addition, for the complex relationship between air pollutants, meteorological factors and ILI cases, Guo et al. found that air pollutants have a significant impact on the mortality due to non-accidental diseases in the short term, and are affected by seasons and rainfall [12]. Furthermore, Tang et al. showed that human respiratory infections were affected by AQI, temperature, humidity and pressure, and discussed the important intervention measures needed to reduce air pollution and thus reduce respiratory infections [20].

In addition, our results reveal that the conditional direct effects of minimum temperature and maximum humidity on the total number of daily reported ILI cases are statistically significant, almost consistent with findings from previous studies [20, 34–37]. The possible biological or mechanistic explanations are as follows: (i) Cold temperatures in winter and low relative humidity, often resulting from indoor heating, increase the frequency of influenza virus transmission. This has been demonstrated by housing infected and uninfected guinea pigs together in an environmental chamber under controlled temperature and humidity conditions [34]. (ii) During the influenza season, a sufficiently high viral load makes hosts more susceptible to the effects of body cooling and/or respiratory tract irritation. In contrast, during non-flu seasons, even with high levels of PM2.5, hosts are less likely to be infected due to lower viral activity. This phenomenon may partly be attributed to the drying effect of breathing in dry air, which can lead to epithelial damage and reduced mucociliary clearance, thereby making hosts more susceptible to respiratory viruses. Conversely, warm temperatures and high humidity appear to enhance host resistance to viral infections, resulting in a lower overall viral load suspended in the air [34, 35]. (iii) Lower absolute humidity levels have been shown to increase influenza infection rates. Experiments using guinea pigs as a transmission model demonstrated that different combinations of temperature and absolute humidity significantly affected transmission rates [37].

There are several novel contributions in our study. Firstly, mediation models were applied to find the complex relationship between meteorological factors, air pollutants and ILI epidemics for the first time. Secondly, we explored the optimal mechanism to describe the mediating effect of air pollutants on the association between meteorological factors and ILI infections by establishing multiple mediation models. Thirdly, through mediation analysis, we can conclude that minimum temperature/maximum humidity moderated by maximum humidity/minimum temperature and pressure directly affect ILI infections, and some of meteorological factors can also indirectly affect ILI infections through air pollutants. Lastly, the rationality of the selected optimal model and the mediating mechanism of air pollution on the association between meteorological factors and ILI infections were verified again based on the daily numbers of reported ILI cases by age $0\sim 4$.

Our study also has several limitations. Firstly, although there are many mediation models and the internal structure of variables is complex, to more accurately and effectively depict their relationships, we only employed six models. Next, to comprehensively study ILI prevalence, additional confounding factors should be considered, such as individual behavioral changes, sex, availability of health facilities, regional economic development, elderly population ratio, and smoking rates, among others. However, data on these factors were not available for collection in this study. Further, only data for Xi’an city in China was included in this study, so our findings were not globally representative. The mediating effect of air pollution may be different for those cities or countries that do not have serious air pollution issues. Lastly, this study provides recommendations for controlling ILI transmission under specific meteorological conditions, such as low temperatures and low humidity during periods of air pollution. It emphasizes the importance of promoting behavioral changes among the public to reduce transmission risks. However, as the study did not account for all potential confounding factors and was conducted in areas with severe air pollution, its findings may not be fully applicable to regions with better environmental conditions. Future studies are needed to overcome these limitations.

## Conclusions

This study presented a novel methodology through studying the underlying mechanism of the association between meteorological factors and ILI infections through assessing the mediating effect of air pollutants by mediation analysis. It demonstrated that meteorological factors minimum temperature/maximum humidity moderated by maximum humidity/minimum temperature and pressure directly affect ILI infections, and that some of meteorological factors can also indirectly affect them through air pollutants. Thus, increasing the maximum humidity and minimum temperature can directly reduce the numbers of ILI cases, or indirectly reduce them by reducing the concentration of air pollutants. Therefore, for mitigating and controlling the ILI prevalence, meteorological forecasts of haze with low temperature, low humidity need to be taken into account, so that the environmental protection departments can take effective control measures to reduce the concentration of air pollutants (such as reducing the exhaust gas emissions of industry and vehicles, reducing the exhaust gas generated by coal-fired heating in northern winter, etc.), the public health departments should advocate that people make behavioral changes, such as avoiding going out or wearing masks when going out and respond positively to control measures.

## Supplementary Information

Supplementary Material 1.

## Data Availability

The data on numbers of reported ILI cases data that support the findings of this study are available from Shaanxi Center for Disease Control and Prevention, but restrictions apply to the availability of these data, which were used under licence for the current study and so are not publicly available. The data are, however, available from the corresponding author upon reasonable request.
