# Supplementary material for: The mediating effect of air pollution on the association between meteorological factors and influenza-like illness in China
Source: BMC Public Health. 2025 Feb 8;25:526. doi: 10.1186/s12889-025-21651-5 (PMC11807337; doi:10.1186/s12889-025-21651-5)
Supplement: Supplementary file 1 — Supplementary Material 1. [file 12889_2025_21651_MOESM1_ESM.pdf]

Supporting Information (SI):The mediating effect of air pollution on the association between meteorological factors and influenza-like illness in China

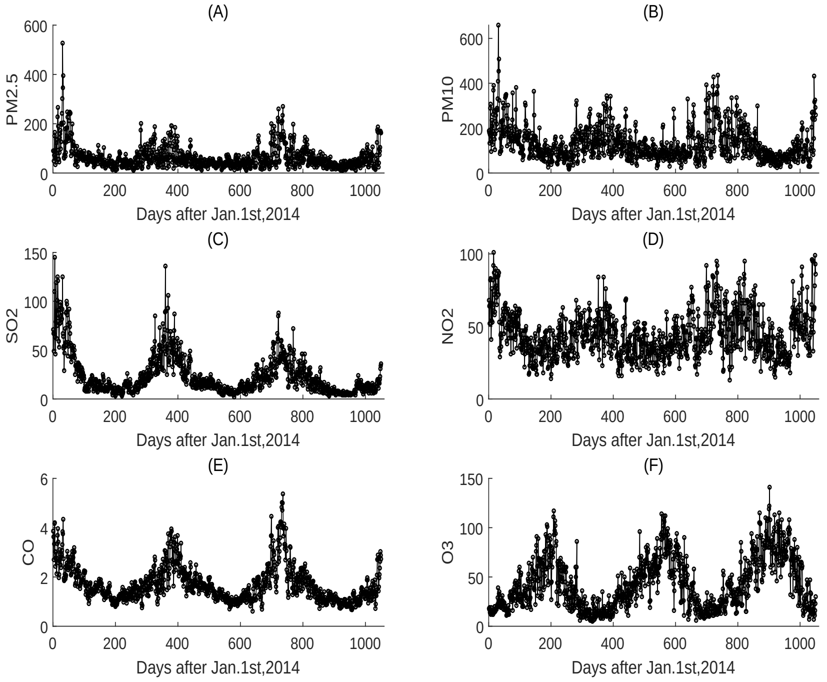

Fig. A.1 Time series of daily data on six air pollutants in Xi'an, Shaanxi Province of China from 1 January 2014 to 15 November 2016.(A) PM25; (B) PM10; (C) SO2; (D) NO2; (E) CO and (F) O3.

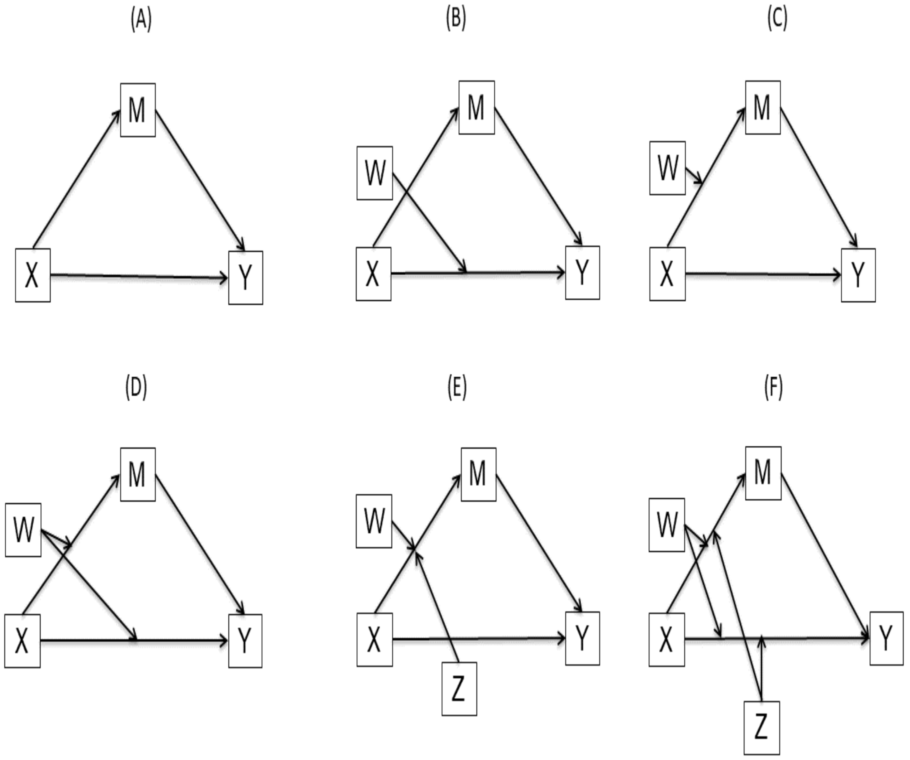

Fig. B.1 Statistical diagrams of the mediation models.

B.0.1 Explanation of the simple mediation model

In model (1), $c^{'}$ describes the direct effect of X on Y, which combines two cases that differ by one unit of X but have values of M, i.e.

$$c^{'} = [\hat{Y}|(X = x + 1, M = m)] - [\hat{Y}|(X = x, M = m)],$$

where m is any value of M, | means conditioned on, $\hat{Y}$ means estimated value of Y based on the model (1). Thus, the positive direct effect means that the higher the X, the higher its effect on Y, while a negative direct effect means that the higher the X, the lower its effect on Y.

The indirect effect of X on Y through M is $a_i \times b_i$. a_i quantifies how much two cases that differ by one unit of X are estimated to differ in their effects M, i.e.

$$a_i = [\hat{M}|(X = x + 1)] - [\hat{M}|(X = x)].$$

The interpretation of b is similar to $c^{'}$, which is

$$b_i = [\hat{Y}|(M = m + 1, X = x)] - [\hat{Y}|(M = m, X = x)].$$

The sign interpretation of a_i and b_i is also analogous to $c^{'}$. The indirect effect shows that two cases that differ by one unit of X are estimated to differ by $a_i \times b_i$ units in their effect on Y as a result of the effect of X on Y, which, in turn, affects Y.

c is used to depict the total effect of X on Y, which quantifies how much two cases that differ by one unit of X are estimated to differ in their effect on Y, i.e.

$$c = [\hat{Y}|(X = x + 1)] - [\hat{Y}|(X = x)].$$

Also c is equal to the sum of the direct and indirect effects of X:

$$c = c^{'} + a_i b_i.$$

B.0.2 Inference about the direct effect of X on Y

The null hypothesis $H_0 : c^{'} = 0$ against the alternative $H_1 : c^{'} \neq 0$. By using the OLS regression program in the PROCESS procedure, the confidence interval of $c^{'}$ can be calculated [2],

$$c^{'}_E - t_{\alpha/2}(n-2)SE_{c^{'}} \leq c^{'} \leq c^{'}_E + t_{\alpha/2}(n-2)SE_{c^{'}},$$

where $c^{'}_E$ is the estimated value for $c^{'}$; $t_{\alpha/2}(n-2)$ is the value of t that cuts off the upper $1 - \alpha/2$ of the $t(n-2)$ distribution from the rest of the distribution; α is the confidence level desired; $SE_{c^{'}}$ is the estimated standard error of $c^{'}$. The lower and upper limit confidence interval of $c^{'}$ are denoted by LLCI and ULCI. The confidence interval involves determining whether an interval estimate for $c^{'}$ includes zero. If the confidence interval does not include 0, the null hypothesis is rejected and then the direct effect is significant; Otherwise, the direct effect is not significant.

B.0.3 Inference about the indirect effect X on Y through M by use of the bootstrap method

The bootstrap method is used to generate an empirically derived representation of the sampling distribution of the indirect effect, and this empirical representation is used for calculation of a confidence interval for $a_i b_i$ [1, 2]. There are six steps for calculation of a confidence interval for $a_i b_i$ as follows:

(i) Take a random sample with sample size of n from the original sample with replacement (n is the size of the original sample), donated by bootstrap sample.

(ii) Estimate the value of a_i and b_i from the model (1), then $a_i \times b_i$ is the estimated indirect effect;

(iii) Repeat step (i) and (ii) k times, and save the value of $a_i \times b_i$ each time (for example, $k = 5000$).

(iv) Sort all of the estimated $a_i \times b_i$ from low to high.

(v) For a given confidence level α, the lower bound of a $(1-100\alpha)\%$ confidence interval is the $(50\alpha)-th$ percentile of the distribution for the value of $a_i \times b_i$ in this distribution of k (i.e. the $0.5k\alpha - th$ position of the sorted distribution).

(vi)The upper bound of a $(1-100\alpha)\%$ confidence interval is the $(100-50\alpha)\%-th$ percentile of the distribution for the value of $a_i \times b_i$ in this distribution of k (i.e. the $[k(1-0.5\alpha)+1]-th$ position of the sorted distribution).

The lower and upper limit confidence interval of $a_i \times b_i$ by use of the bootstrap method are denoted by BootLLCI and BootULCI. The confidence interval involves determining whether an interval estimate for $a_i \times b_i$ includes zero or not. If the confidence interval does not include 0, the null hypothesis is rejected and then the indirect effect is significant; Otherwise, the indirect effect is not significant.

B.0.4 Inference about the total effect of X on Y

The null hypothesis $H_0 : c = 0$ against the alternative $H_1 : c \neq 0$. By using the OLS regression program in the PROCESS procedure, the confidence interval of c can be calculated [2],

$$c_E - t_{\alpha/2}(n-2)SE_c \leq c \leq c_E + t_{\alpha/2}(n-2)SE_c,$$

where c_E is the estimated value for c; $t_{\alpha/2}(n-2)$ is the value of t that cuts off the upper $1-\alpha/2$ of the $t(n-2)$ distribution from the rest of the distribution; $SE_{c'}$ is the estimated standard error of c. The lower and upper limit confidence interval of c are denoted by LLCI and ULCI. The confidence interval involves determining whether an interval estimate for c includes zero or not. If the confidence interval does not include 0, the null hypothesis is rejected and then the total effect is significant; Otherwise, the total effect is not significant.

References

[1] Fang J, Zhang MQ, Li XP (2011) Estimating confidence intervals of mediating effects by using the distribution of the product, bootstrap and markov chain monte carlo methods. Advances in Psychological Science 19(5):765–774.

[2] Hayes AF (2017) Introduction to mediation, moderation, and conditional process analysis: A regression-based approach. Guilford publications

Table B.1 The mediating effects of other air pollutants for model (6)($Y = Y_1$).

	$R^2(X \to M)$ (Yes/No)	$R^2(M \to Y)$ (Yes/No)	$R^2(X \to Y)$ (Yes/No)	Direct effect (Conditional direct effects) (Yes/No)	Indirect effect (Conditional indirect effects)(Yes/No)	Total effect (Yes/No)	Index of moderated mediation (Yes/No)
model (6) ($M = PM2.5$, $X = X_3$, $W = X_5$, $Z = X_7$)	0.2919(Yes)	0.3845(Yes)	/	Yes	No	/	W(No),Z(No)
model (6) ($M = PM10$, $X = X_3$, $W = X_5$, $Z = X_7$)	0.3333(Yes)	0.3857(Yes)	/	Yes	No	/	W(No),Z(No)
model (6) ($M = SO_2$, $X = X_3$, $W = X_5$, $Z = X_7$)	0.5586(Yes)	0.3930(Yes)	/	Yes	Yes	/	W(No),Z(No)
model (6) ($M = NO_2$, $X = X_3$, $W = X_5$, $Z = X_7$)	0.2845(Yes)	0.3883(Yes)	/	Yes	Yes	/	W(Yes),Z(Yes)
model (6) ($M = CO$, $X = X_3$, $W = X_5$, $Z = X_7$)	0.5727(Yes)	0.3845(Yes)	/	Yes	No	/	W(No),Z(No)
model (6) ($M = O_3$, $X = X_3$, $W = X_5$, $Z = X_7$)	0.6424(Yes)	0.3894(Yes)	/	Yes	Yes	/	W(No),Z(Yes)
model (6) ($M = PM2.5$, $X = X_5$, $W = X_3$, $Z = X_7$)	0.2822(Yes)	0.3658(Yes)	/	Yes/No	No	/	W(No),Z(No)
model (6) ($M = PM10$, $X = X_5$, $W = X_3$, $Z = X_7$)	0.3033(Yes)	0.3654(Yes)	/	Yes/No	No	/	W(No),Z(No)
model (6) ($M = SO_2$, $X = X_5$, $W = X_3$, $Z = X_7$)	0.5684(Yes)	0.3736(Yes)	/	Yes/No	Yes/No	/	W(Yes),Z(Yes)
model (6) ($M = NO_2$, $X = X_5$, $W = X_3$, $Z = X_7$)	0.2737(Yes)	0.3670(Yes)	/	Yes/No	No	/	W(No),Z(No)
model (6) ($M = CO$, $X = X_5$, $W = X_3$, $Z = X_7$)	0.5743(Yes)	0.3655(Yes)	/	Yes/No	No	/	W(No),Z(No)
model (6) ($M = O_3$, $X = X_5$, $W = X_3$, $Z = X_7$)	0.6054(Yes)	0.3782(Yes)	/	Yes/No	Yes/No	/	W(Yes),Z(Yes)

Note: Yes/No in brackets indicates whether the indicator is statistically significant. Y_1 denote the total number of daily reported ILI cases. We use X_3 for minimum temperature, X_5 for maximum humidity, X_7 for pressure, respectively.

Table B.2 The mediating effects for models (1-6)($Y = Y_2$, $M = AQI$).

	$R^2(X \to M)$ (Yes/No)	$R^2(M \to Y)$ (Yes/No)	$R^2(X \to Y)$ (Yes/No)	Direct effect (Conditional direct effects) (Yes/No)	Indirect effect (Conditional indirect effects)(Yes/No)	Total effect (Yes/No)	Index of moderated mediation (Yes/No)
model (1) ($X = X_3$)	0.1991(Yes)	0.2345(Yes)	0.2340(Yes)	Yes	No	Yes	/
model (1) ($X = X_5$)	0.0069(Yes)	0.0713(Yes)	0.0390(Yes)	Yes	Yes	Yes	/
model (1) ($X = X_7$)	0.0622(Yes)	0.1748(Yes)	0.1654(Yes)	Yes	Yes	Yes	/
model (1) ($X = X_{10}$)	0.0459(Yes)	0.0389(Yes)	0.0047(Yes)	No	Yes	Yes	/
model (2) ($X = X_3$, $W = X_5$)	0.1991(Yes)	0.2494(Yes)	/	Yes	No	/	/
model (2) ($X = X_3$, $W = X_7$)	0.1991(Yes)	0.2545(Yes)	/	Yes	No	/	/
model (2) ($X = X_3$, $W = X_{10}$)	0.1991(Yes)	0.2404(Yes)	/	Yes	No	/	/
model (2) ($X = X_5$, $W = X_3$)	0.0069(Yes)	0.2494(Yes)	/	Yes/No	No	/	/
model (2) ($X = X_5$, $W = X_7$)	0.0069(Yes)	0.2002(Yes)	/	Yes	Yes	/	/
model (2) ($X = X_5$, $W = X_{10}$)	0.0069(Yes)	0.0800(Yes)	/	Yes	Yes	/	/
model (3) ($X = X_3$, $W = X_5$)	0.2471(Yes)	0.2345(Yes)	/	Yes	No	/	No
model (3) ($X = X_3$, $W = X_7$)	0.2493(Yes)	0.2345(Yes)	/	Yes	No	/	No
model (3) ($X = X_3$, $W = X_{10}$)	0.2324(Yes)	0.2345(Yes)	/	Yes	No	/	No
model (3) ($X = X_5$, $W = X_3$)	0.2471(Yes)	0.0713(Yes)	/	Yes	Yes	/	Yes
model (3) ($X = X_5$, $W = X_7$)	0.0907(Yes)	0.0713(Yes)	/	Yes	Yes/No	/	Yes
model (3) ($X = X_5$, $W = X_{10}$)	0.0648(Yes)	0.0713(Yes)	/	Yes	Yes/No	/	No
model (4) ($X = X_3$, $W = X_5$)	0.2471(Yes)	0.2494(Yes)	/	Yes	No	/	No
model (4) ($X = X_3$, $W = X_7$)	0.2493(Yes)	0.2545(Yes)	/	Yes	No	/	No
model (4) ($X = X_3$, $W = X_{10}$)	0.2324(Yes)	0.2404(Yes)	/	Yes	No	/	No
model (4) ($X = X_5$, $W = X_3$)	0.2471(Yes)	0.2494(Yes)	/	Yes/No	No	/	No
model (4) ($X = X_5$, $W = X_7$)	0.0907(Yes)	0.2002(Yes)	/	Yes	Yes/No	/	Yes
model (4) ($X = X_5$, $W = X_{10}$)	0.0648(Yes)	0.0800(Yes)	/	Yes	Yes/No	/	No
model (5) ($X = X_3$, $W = X_5$, $Z = X_7$)	0.2917(Yes)	0.2345(Yes)	/	Yes	No	/	W(No),Z(No)
model (5) ($X = X_3$, $W = X_{10}$, $Z = X_7$)	0.2868(Yes)	0.2345(Yes)	/	Yes	No	/	W(No),Z(No)
model (5) ($X = X_5$, $W = X_3$, $Z = X_7$)	0.2779 (Yes)	0.0713(Yes)	/	Yes	Yes/No	/	W(Yes),Z(No)
model (5) ($X = X_5$, $W = X_{10}$, $Z = X_7$)	0.1262(Yes)	0.0713(Yes)	/	Yes	Yes/No	/	W(No),Z(Yes)
**model (6)** ($X = X_3$, $W = X_5$, $Z = X_7$)	**0.2917(Yes)**	**0.2628(Yes)**	/	**Yes**	**No**	/	**W(No),Z(No)**
model (6) ($X = X_3$, $W = X_{10}$, $Z = X_7$)	0.2868(Yes)	0.2567(Yes)	/	Yes	No	/	W(No),Z(No)
**model (6)**($X = X_5$, $W = X_3$, $Z = X_7$)	**0.2779 (Yes)**	**0.2728(Yes)**	/	**Yes/No**	**No**	/	**W(No),Z(No)**
model (6) ($X = X_5$, $W = X_{10}$, $Z = X_7$)	0.1262(Yes)	0.2024(Yes)	/	Yes	Yes/No	/	W(No),Z(Yes)

Note: Yes/No in brackets indicates whether the indicator is statistically significant.Y_2 denote the number of daily reported ILI cases by age $0 \sim 4$. We use Z_1 for AQI, X_3 for minimum temperature, X_5 for maximum humidity, X_7 for pressure, X_{10} for maximum wind speed, respectively.
